# The Role of EBV-Encoded LMP1 in the NPC Tumor Microenvironment: From Function to Therapy

**DOI:** 10.3389/fonc.2021.640207

**Published:** 2021-02-25

**Authors:** Angela Kwok-Fung Lo, Christopher W. Dawson, Hong Lok Lung, Ka-Leung Wong, Lawrence S. Young

**Affiliations:** ^1^ Department of Chemistry, Hong Kong Baptist University, Hong Kong, China; ^2^ Warwick Medical School, University of Warwick, Coventry, United Kingdom; ^3^ Department of Biology, Hong Kong Baptist University, Hong Kong, China

**Keywords:** Epstein-Barr virus, nasopharyngeal carcinoma, latent membrane protein 1, tumor microenvironment, therapy

## Abstract

Nasopharyngeal carcinoma (NPC) is closely associated with Epstein-Barr virus (EBV) infection. It is also characterized by heavy infiltration with non-malignant leucocytes. The EBV-encoded latent membrane protein 1 (LMP1) is believed to play an important role in NPC pathogenesis by virtue of its ability to activate multiple cell signaling pathways which collectively promote cell proliferation and survival, angiogenesis, invasiveness, and aerobic glycolysis. LMP1 also affects cell-cell interactions, antigen presentation, and cytokine and chemokine production. Here, we discuss how LMP1 modulates local immune responses that contribute to the establishment of the NPC tumor microenvironment. We also discuss strategies for targeting the LMP1 protein as a novel therapy for EBV-driven malignancies.

## Introduction

Nasopharyngeal Carcinoma (NPC) is a malignancy arising in mucosal epithelium of the nasopharynx. NPC is a rare cancer in western countries but has high rates of incidence throughout Southeast Asia, southern China, and the Mediterranean basin. According to the International Agency for Cancer, approximately 129,000 new cases of NPC were reported globally in 2018. More than 70% of the new cases are reported in Southern China and Southeast Asia, while only 1.7% of cases are found in the USA (1).

According to the World Health Organization (WHO), NPC is classified into three histopathological subtypes: type I keratinizing squamous cell carcinoma, type II non-keratinizing squamous cell carcinoma and type III basaloid squamous carcinoma. Type II non-keratinizing NPC is subdivided into differentiated and undifferentiated tumors (2). The keratinizing NPC is relatively common in the Western countries (~75%), while the non-keratinizing tumors account for more than 97% of NPC cases in Southern China and Southeast Asia. The non-keratinizing form of NPC is strongly associated with EBV infection regardless of geographical distribution, ethnic origin and local prevalence of the disease. EBV is therefore believed to be a critical etiological factor in NPC pathogenesis (3–5).

### EBV Infection in the Host

The association of NPC with EBV was firstly discovered by sero-epidemiological studies. Elevated IgA antibody titers to several EBV replicative antigens were consistently detected in the serum of NPC patients (3, 6). Subsequently, elevated levels of circulating cell-free EBV DNA were detected in NPC patients. Since EBV DNA levels show a strong correlation with NPC tumor stage and overall survival, the detection of circulating cell-free EBV DNA has become a gold standard biomarker (7). In NPC tissues, clonal EBV genomes and EBV-encoded latent gene products are consistently detected. While EBV can be detected in invasive carcinoma and high-grade dysplastic lesions, low-grade dysplasia and normal nasopharyngeal epithelium are uniformly negative for EBV. These findings imply that latent viral infection occurs before the clonal expansion of tumor cells and that early genetic alterations may predispose precancerous cells to EBV latent infection. The expression of EBV-encoded latent viral genes may confer a growth and survival advantage, facilitating the expansion of genetically altered EBV-infected clones. EBV can efficiently transform resting B cells *in vitro*, an effect that relies on virus latent gene expression and that models the ability of the virus to contribute to various B cell malignancies *in vivo* (3, 6, 8).

EBV is a ubiquitous virus that infects more than 95% of the population worldwide. Primary EBV infection in immune-competent hosts (naïve hosts), stimulates a vigorous cytotoxic T cell (CTL) response against latent and lytic antigens, which controls the expansion of EBV-transformed B cells. EBV establishes lifelong latent infections in the memory B cell pool that circulates between the blood and pharyngeal lymphoid tissues. Periodic switching from a latent to a lytic infection within the oropharynx, in both B cells and/or epithelial cells, results in virus shedding in the throat (9, 10).

In most individuals, both primary infection and long-term virus carriage are asymptomatic; however, EBV is linked to a number of human malignancies. In addition to NPC, EBV infection is linked with endemic Burkitt’s lymphoma (BL), Hodgkin’s lymphoma (HL), post-transplant lymphoproliferative disease (PTLD) and a proportion of gastric carcinoma (GC) (3, 6). During EBV latent infection, EBV adopts various forms of latency (Latency I, II and III), which differ in their repertoire of latent genes expressed. *In vitro*, EBV efficiently infects and transforms primary B cells to generate immortalized lymphoblastoid cell lines (LCLs). Both LCLs and PTLD display type III latency, where all latent genes including nuclear proteins (EBNA-1, -2, -3a/3b/c, and -LP), membrane proteins (LMP-1, -2a, -2b), and non-coding RNAs (EBER1, EBER2 and BART RNAs; BHRF1 and BART miRNAs) are expressed. While, HL and NPC display a type II latency, with the expression of EBNA1, LMP1, LMP2a/2b, EBER1/2 RNAs and BART RNAs and miRNAs. Endemic BL predominantly display a type I latency program, expressing EBNA1, LMP2a/2b, EBER1/2 RNAs and BART RNAs and miRNAs (6, 9, 11).

### LMP1 Expression in NPC

Among the EBV-encoded gene products expressed in NPC, latent membrane protein 1 (LMP1) is of particular interest as it displays oncogenic properties *in vivo* and *in vitro*. LMP1 protein expression in NPC tissues is variable. By traditional chromogenic immunohistochemical (IHC) methods, the detection of LMP1 in NPC cases varies between 20%–60%. However, LMP1 protein is detected in almost all premalignant or preinvasive NPC tissue samples (4, 12), suggesting the contribution of LMP1 during the early stage of NPC pathogenesis. Interestingly, LMP1 positive tumors are found to be more aggressive than LMP1 negative tumors and prone to invade lymph nodes (13, 14). LMP1 expression is also significantly correlated with the expression of proteins that mediate invasion, angiogenesis and metastasis, such as Twist, MMP9, c-Met, Ets-1 in NPC tumors (14–16). In addition, LMP1-positive NPC tissues are significantly associated with lymph node metastasis and poor overall survival (OS). LMP1 is also a strong risk factor for poor prognosis in NPC (16–18). It is now accepted that LMP1 is a potent cancer driver, being involved at different stages in NPC progression. However, the lack of LMP1 protein expression in some NPC samples has raised questions about the contribution of LMP1 in NPC development. While this question is still controversial, a recent study comparing a sensitive immunofluorescence staining method with conventional IHC methodologies confirmed the presence of LMP1 protein in all primary NPC samples, with weak to moderate levels of staining. This contrasted with traditional IHC staining, where a complete lack or only weak level of LMP1 protein expression was observed on the same NPC tissues (12). These findings suggest that very low levels of LMP1 protein expression, which are undetectable by traditional IHC methods may be sufficient to elicit biological effects and to promote tumor progression. Indeed, our previous study has shown that very low levels of LMP1, which are virtually undetectable, are sufficient to activate NFκB signaling, a key pathway contributing to LMP1-mediated cell growth and survival (19). In contrast, high levels of LMP1 expression in NPC cells induced growth inhibition and also increased sensitization to cisplatin-induced apoptosis (20). Therefore, low levels of LMP1 expression in NPC cells may be critical for LMP1 to mediate its oncogenic and metastatic effects.

### LMP1-Mediated Signaling Pathways and Their Associated Oncogenic Effects

**Figure 1:**
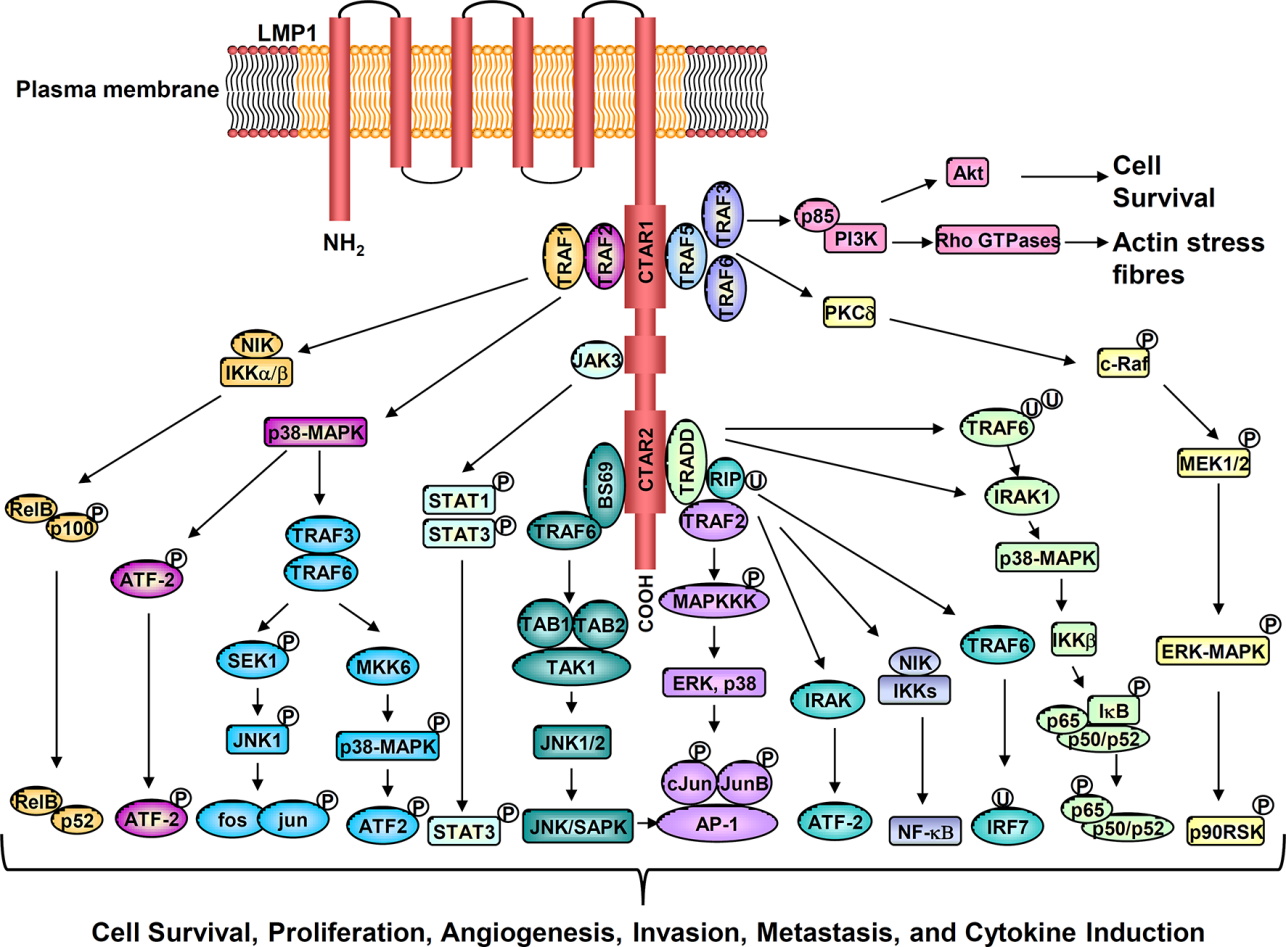
Activation of multiple cell signaling pathways by LMP1 promotes cell survival, proliferation, angiogenesis, invasion, metastasis, and cytokine production.

LMP1 is an integral membrane protein comprising a short N-terminal cytoplasmic domain, six transmembrane spanning loops, and a long C-terminal cytoplasmic tail which contains two signaling domains designated c-terminal activating regions, CTAR-1 and CTAR-2. LMP1 functions as a constitutively active viral mimic of CD40, a member of the tumor necrosis factor (TNF) receptor superfamily (8). Through its two c-terminal activating regions (CTAR regions), LMP1 activates a number of signaling cascades. These include NF-κB, PI3K–AKT, ERK–MAPK, JNK, JAK-STAT, and the p38/MAPK signaling pathways. Collectively, these pathways coordinate the expression of a variety of downstream cellular targets that contribute to the oncogenic activity of LMP1 (**Figure 1**). The absolute requirement for LMP1 in EBV-driven B cell immortalization, the induction of lymphomagenesis and epidermal hyperplasia in LMP1 transgenic mice, and the malignant transformation of established rodent fibroblast cell lines, underscores the oncogenic potential of the protein. Consistent with this oncogenic function, LMP1 has been shown to exert a variety of pleiotropic effects which appear to be context dependent. These include the suppression of senescence in mouse embryo fibroblasts; a blockade of epithelial cell differentiation; the induction of an epithelial-mesenchymal transition (EMT), cell proliferation and anchorage-independent growth; upregulation of anti-apoptotic proteins to increase cell survival; the induction of angiogenic and metastatic factors to promote increase cell motility and invasive growth; and dysregulated energy metabolism. LMP1 also mediates many immunomodulatory effects by inducing a set of immune-associated proteins, to shield virus infected tumor cells from immune attack (8, 21, 22).

In this review, we describe the pathological effects of LMP1 in NPC, focusing on its contribution to the formation and maintenance of the tumor microenvironment (TME). We will also discuss potential therapeutics that aim to target the LMP1 protein or to antagonize the immunosuppressive TME in NPC.

## The Interaction of Stromal Cells With EBV-Infected NPC Cells in the Tumor Microenvironment

In healthy EBV-seropositive individuals, CD8+ T cell responses are only weakly directed against LMP1. Instead, they are polarized towards specific EBV lytic antigens and the EBNA3 family of proteins (3a,3b,3c) (10). EBV persistence in resting memory B cells is immunologically silent as cells fail to elicit a robust immune response. However, viral reactivation and growth transformation are subject to control by cytotoxic T lymphocytes (CTL) and antibodies specific for both lytic and latent EBV antigens (23). As far as is known, NPC patients are immunocompetent and do not succumb to EBV-induced lymphoproliferative disease usually associated with extreme immunosuppression. Circulating CD4+ and CD8+ memory T cells specific for the tumor-associated EBV proteins (EBNA1, LMP1 and LMP2) are mostly intact and functional, although their frequencies are somewhat lower than that observed in healthy donors (6, 9, 10, 24). In contrast, natural killer (NK) cells isolated from the NPC patients can be efficiently activated and expanded to exert cytolytic activity (25). However, the EBV-specific immune response in NPC patients appears incapable of eliminating tumor cells.

### Lymphocyte Infiltration in NPC

In addition to the consistent presence of EBV, NPC is characterized by the presence of CD45+ tumor-infiltrating leukocytes (TILs) within the tumor mass, leading to NPC’s original classification as a lymphoepithelioma. The tumor usually contains a heavy infiltrate of EBV-negative non-malignant leukocytes that are primarily composed of CD8+ and CD4+ T cells and subsets of natural killer (NK) cells, B cells, mast cells and various types of myeloid cells including monocytes, macrophages, and neutrophils. Several infiltrating cell types with known immunosuppressive function are present, including CD4+ CD25+Foxp3+T regulatory cells (Tregs), myeloid-derived suppressor cells (MDSCs) and tumor-associated macrophages (TAMs) (26, 27). Although TILs account for a large fraction of stromal cells in the tumor microenvironment (TME), they seem to have no inhibitory effects on NPC tumor cells. Studies have revealed that infiltrating EBV-specific CTLs isolated from NPC biopsies lack cytotoxic activity and fail to produce IFN-*γ* in response to stimulation with autologous EBV immortalized lymphoblastoid cell line (LCL) (28). These findings suggest that the presence of abundant of TILs within the TME cannot suppress viral infection or tumor growth and may inadvertently fuel tumor growth and progression through a number of mechanisms. TILs are likely influenced by cytokines, immune checkpoint proteins and exosomes secreted by cancer cells.

### Immunosuppressive Effects of LMP1 Epitopes

Studies have shown that LMP-positive NPC tumors contain significantly larger lymphoid infiltrates than LMP1-negative cases (29, 30), implicating a role for LMP1 in promoting lymphocyte infiltration into the NPC TME. However, LMP1 is poorly immunogenic, generating weak humoral and cellular immune responses. In healthy EBV-seropositive individuals, CD8+ T cell responses are not generally directed against LMP1. Instead, responses are directed towards other EBV lytic antigens and the strongly immunogenic EBNA3 family of proteins (31). The lack of immunogenicity of LMP1 may be associated with the immunosuppressive effects of LMP1 protein epitopes. Previous studies have shown that purified LMP1 protein or a peptide (LALLFWL) derived from the first transmembrane of LMP1 stimulate inhibitory T cells (so-called regulatory T cells or Tregs) to secrete high levels of IL-10, which inhibited mitogen, antigen and CD3/CD28-stimulated T effector cell proliferation, NK cell cytotoxicity and antigen-induced IFN-*γ* secretion (32, 33). It appears, therefore, that both CD4+ Th1 and CD8+ cytotoxic responses against LMP1 and other tumor antigens are repressed in NPC contributing to an immunosuppressive TME that not only favors viral persistence, but also supports tumor cell growth and survival.

### NF-κB & STAT3 Signal Activation by LMP1

Chronic infection and inflammation are believed to aid cancer development. EBV infection of nasopharyngeal epithelial cells activates the NF-κB and STAT3 pathways, leading to an increase in the secretion of many inflammatory cytokines and chemokines. These, in turn, recruit a large immune infiltrate, which can inadvertently promote tumor progression (34, 35).

NF-κB and STAT3 are two essential signaling pathways involved in shaping the TME. Through the induction of pro-inflammatory cytokines and chemokines, tumor cells can interact with and influence the cellular composition of the TME. Our previous studies have shown that LMP1 alone, or EBV latent infection, dramatically upregulates the NF-κB and STAT3 signaling pathways in nasopharyngeal epithelial cells (35, 36). These findings are corroborated by studies performed on primary NPC tumors, where constitutively active NF-κB and STAT3 signaling pathways are commonly detected in tumor cells (35, 37, 38). Interestingly, genomic analysis indicates that somatic mutations that drive aberrant NF-κB activation in NPC are mutually exclusive with the expression of LMP1. LMP1 activates NF-κB through its CTAR1 and CTAR2 domains by engaging TRAFs, TRADD, and RIP. LMP1 induces both canonical IKK-dependent and non-canonical NIK-dependent NF-κB pathways (8, 38) (**Figure 1**). The NF-κB family of transcription factors influence a variety of cellular processes, ranging from cell proliferation, apoptosis, oncogenesis, and inflammation. Accordingly, NF-κB activation mediated by either LMP1 or intrinsic somatic mutations appear essential for NPC progression.

## LMP1 Modulates the Response of Stromal Cells in the Tumor Microenvironment to Promote Cancer Progression

**Figure 2:**
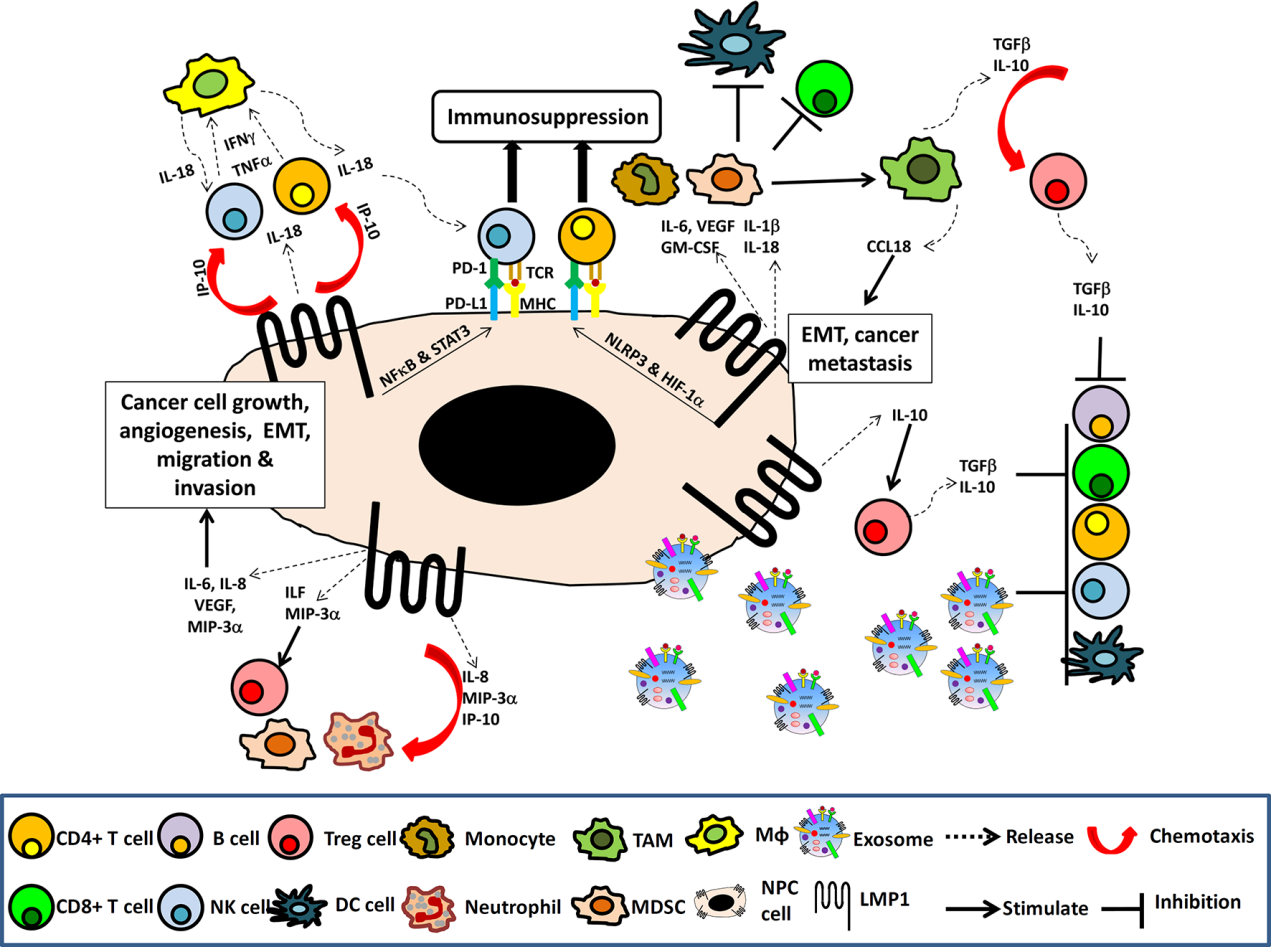
The NPC immune microenvironment. Schematic illustrating the induction of NF-κB and STAT3 signaling pathways and multiple immune cytokines by LMP1 in cancer cells resulting in cancer cell growth, angiogenesis, EMT, migration and invasion as well as chemotaxis of lymphocytes, NK cells, macrophage (Mφ), neutrophil, MDSC, Tregs, to the tumor site. The presence of TGFβ and IL-10, along with the LMP1 upregulation of PDL-1 and PD-1, and the release of LMP1-containing exosomes into the tumor TME to induce expansion of Tregs, MDSC and TAM, which create an immunosuppressive TME that facilitates tumor evasion.

Through activation of the NF-κB, STAT3 and additional signaling pathways, LMP1 can induce the expression of multiple downstream targets involved in chronic inflammatory responses (**Figure 1**). These include a variety of interleukins (IL-1, IL-6, IL-8, IL-10 IL-18, TNF-α), chemokines (MCP-1/CCL2, MIP-1α/CCL3, MIP-1β/CCL4, RANTES/CCL5, MIP-3α/CCL20, MDC/CCL22), IP-10/CXCL10; adhesion molecules (LFA, ICAM-1, CD18); antigen processing and presentation proteins (MHC class I, II and TAP), cyclooxygenase-2 (COX-2), vascular endothelial growth factor (VEGF), Hypoxia-inducible factor 1-α (HIF-1α), all of which are involved in immune evasion, cell growth, glycolysis, angiogenesis, invasion, metastasis, and epithelial-mesenchymal transition (EMT) (**Figure 2**) (8, 31, 39, 40).

### LMP1 Induction of Immunomodulatory Molecules Generates an Inflammatory Infiltrate in the NPC TME

When expressed in NPC cells, LMP1 can stimulate the expression of numerous immunomodulatory molecules. Many of these factors are potent chemoattractants, which recruit both T cells and macrophages to the TME. Members of this large family include LIF, IL-6, IL-8, IP-10, MDC, MIP-1α, MIP-1β, MIP-3α, NAP-2, RANTES and pan-GROs (40). Interestingly, many of these cytokines, including LIF, IL-6, IL-8, IP-10 and TNFα, VEGF and MIP-3α, are found at high levels in the plasma of NPC patients compared to healthy controls. Studies have shown that high levels of IL-6, IL-8, IP-10 and MIP-3α, were found to correlate with EBV DNA load in advanced NPC patients, while patients with the elevated levels of IL-8, VEGF, MIP-3α, in addition to high EBV DNA loads, had a worse prognosis (35, 41–43).

IL-6 is a pro-inflammatory cytokine that functions as a growth factor for cancer cells. IL-6, VEGF, MIP-3α, all of which are induced by LMP1, have been shown to promote epithelial cell growth, cell migration and invasion (44–47). IL-8 was discovered as a leukocyte chemoattractant and subsequently found to play roles in cancer progression. Tumor-derived IL-8 can induce cancer cell proliferation, an epithelial-to-mesenchymal transition (EMT) and cell migration (40, 48). IL-8, MIP-3α and IP-10 can recruit neutrophils, other immune cells and immunosuppressors such as MDSCs and Tregs, to tumor sites (26, 49, 50). Furthermore, LIF and MIP-3α can exert immunosuppressive effects through activation and expansion of Tregs (51, 52). VEGF has been shown to recruit monocytes to NPC tumor sites where monocytes can be induced to differentiate into TAMs through the action of GM-CSF. The secretion of CCL18 by activated TAMs can promote EMT and NPC metastasis (53). Increased macrophage infiltration within the tumor has been found to correlate with poor survival in NPC patients (54, 55). Collectively, these findings suggest that LMP1 can drive NPC progression by stimulating the expression of many immunomodulatory molecules, which creates a TME with a chronic inflammatory infiltrate.

### LMP1 Induction of IL-18 & IP-10 Recruit and Suppresses the Activity of CXCR3+ NK Cells and T Cells

A study by Hu et al. revealed that both tumor cells and CD68+ macrophages in NPC tissues expressed IL-18; Moreover, they found that CD3+ T cells and CD94+ NK cells in the TME expressed IFN-*γ* (56). Another study by Teichmann et al. revealed that expression of IP-10/CXCL10, an IFN-*γ* inducible protein, was increased in tumor cells of NPC tissues in which numerous CXCR3 expressing lymphocytes were present (57).

IL-18 is a downstream target of LMP1 (58). IL-18 was initially identified as a pro-inflammatory cytokine, that functions to enhance Th1 responses by promoting IFN-*γ* secretion from NK and T cells. IP-10/CXCL10 is a chemoattractant for CXCR3+ T cells and macrophages. IP-10/CXCL10 expression is induced by IFN-*γ* and TNF-α and can also be induced by LMP1 through activation of the NF-κB signaling pathway (57). Accordingly, IL-18 secreted by LMP1 expressing NPC cells stimulates infiltrating T cells and NK cells to produce IFN- *γ*, that consequently activates CD68+ macrophages to secrete IL-18 reciprocally initiating a positive feedback loop of leukocyte recruitment. On the other hand, CXCL-10 secretion by both LMP1-expressing NPC cells and IFN-*γ* activated NPC cells promotes recruitment of additional CXCR3+ T cells and macrophages into the TME, further increasing lymphocyte infiltration.

Based on this mechanism, it is believed that IL-18 and IP-10/CXCL10 do not exert tumor-suppressive effects, but rather, promote IFN-*γ* secretion and chemoattraction of CXCR3+ cells into the NPC TME. CXCR3+ T cells and NK cells recruited to the TME by IL-18 and IP-10/CXCL10 are unlikely to possess anti-tumor function, although they are active in IFN*γ* production. Indeed, tumor-derived IL-18 has been found to promote tumor progression, despite its pro-inflammatory function. High expression of IL-18 has been detected in various cancers, and tumor-derived IL-18 is associated with a poor prognosis (59, 60). In NPC, the elevated levels of IL-18 in cancer cells were associated with a high density of tumor infiltrating NK cells and poor overall rates of survival (61). IL-18 had been shown to exert potential immunosuppressive effects in cancer by increasing expression of programmed cell death-1 (PD-1) on activated mature NK cells (59, 61). Therefore, IL-18 and IP-10/CXCL10 in NPC may play dual roles in the recruitment and suppression of CXCR3+ tumor infiltrating NK cells and T cells (**Figure 2**).

### LMP1-Mediated PD-1/PD-L1 Pathway Suppresses the Anti-Tumor Activities of CXCR3+ NK Cells and T Cells

PD-1 is an inhibitory receptor (also known as a maker of functional exhaustion) expressed on activated TILs. PD-1 functions by binding its ligand, programmed cell death ligand 1 (PD-L1), which is commonly expressed on tumor cells and immune cells. PD-1 and PD-L1 belong to a family of immune checkpoint proteins that can inhibit the proliferation and induce apoptosis of immune cells, leading to immune suppression. Several IHC-based studies have identified PD-L1 expression on tumor cells and PD-1 on TILs in NPC tissues. High expression of PD-L1 in NPC tissues was significantly associated with poor overall survival, with co-expression of PD-1 and PD-L1 in NPC tissues showing the poorest overall survival (9, 62–64). Interestingly, LMP1 has been demonstrated to upregulate PD-L1 in NPC cells through the MAPKs/AP-1, JAK3/STAT3 and NF-κB pathways. Similarly, the expression of PD-L1 in NPC cells can be induced by IFN-*γ* activated JAK-STAT pathway. Upon IFN-*γ* treatment, LMP1-positive NPC tumor cells could be induced to express higher levels of PD-L1 than LMP1-negative NPC cells, indicating a synergistic effect between LMP1 and IFN-*γ* in the regulation of PD-L1 (64). Collectively, these findings suggest that IL-18 induced by LMP1-expressing NPC cells promote IFN-*γ* secretion and PD-1 expression on CXCR3+ T and NK cells. On the other hand, LMP1 and IFN-*γ* upregulate PD-L1 expression on cancer cells. Therefore, co-expression of PD-1 and PD-L1 in TME suppress local effector T cell anti-tumor function (**Figure 2**). Therefore, targeting the PD-L1/PD1 pathway to interrupt immune suppression may be a promising anti-tumor strategy and will be further discussed later in this review.

### LMP1-Mediated Glycolysis Facilitates Cancer Development and Tumor Evasion

We and others have shown that LMP1 is capable of promoting aerobic glycolysis to enhance malignant transformation, tumor progression and resistance to radiotherapy. LMP1 promotes aerobic glycolysis by upregulating c-Myc, HIF-1α and FGF2/FGFR1 signaling, which collectively induce the expression of pyruvate kinase M2 (PKM2), lactate dehydrogenase A (LDHA), hexoinase2 (HK2) and the glucose transporter 1 (GLUT-1). LMP1-mediated aerobic glycolysis increased cell uptake of glucose and glutamine, enhanced LDHA activity and lactate production, facilitating rapid cell proliferation by providing energy as well as metabolic intermediates for the anabolic biosynthesis of macromolecules. The acidic TME generated by increased lactate production also promotes tumor cell migration and invasion (65, 66).

In a recent study, Cai et al. demonstrated that the expression of LMP1 in tumor cells correlated with the expression of GLUT-1, and an abundance of CD33+ myeloid-derived suppressor cells (MDSCs) in NPC tissues. High levels of LMP1 in NPC were associated with poor disease-free survival (DFS). Also, a large abundance of CD33+ MDSCs was found to be a reliable indicator of poor prognosis (67). MDSCs are immature myeloid cells which are recruited to the inflammatory and hypoxic tumor site under the influence of many chemokines and cytokines. In the TME, infiltrating MDSCs rapidly differentiate into tumor-associated macrophages (TAMs) and produce a large number of immunosuppressive cytokines such as TGF-β and IL-10 to attract Tregs to the TME. MDSCs also inhibit the antigen presentation function of DC cells and the anti-tumor function of cytotoxic TILs (68). Cai et al. showed that aerobic glycolysis induced by LMP1 in NPC cells upregulated COX-2 expression and activation of the NFκB pathway (p65 phosphorylation), which increased IL-6 and GM-CSF secretion. LMP1-mediated glycolysis also activated the Nod-like receptor family 3 (NLRP3) inflammasome pathway, which increased IL-1β and IL-18 production. Induction of IL-6, IL-1β, IL-18 and GM-CSF in the TME subsequently promoted MDSC differentiation and expansion as well as proliferation and apoptotic resistance of EBV-infected NPC cells (67, 69, 70). Besides, NLRP3 inflammasome and HIF-1a have been found to upregulate PD-L1 expression, in turn blocking effector immune cell proliferation (71, 72). Therefore, LMP1-mediated glycolytic activities suppress anti-tumor immune responses in NPC through multiple pathways.

### LMP1 Enhances the Immunosuppressive Function of Regulating T Cells By Induction of IL-10

IL-10 is an immunosuppressive cytokine commonly expressed in NPC tumor cells. High levels of IL-10 correlate positively correlated with low numbers of CTLs in NPC. IL-10 modulates the immune response by suppressing the activity of antigen-presenting cells (APC) and the inhibitory function of T helper type 1 (Th1) cells and CTLs. Therefore, the presence of IL-10 in NPC tissues promotes local immunosuppression within the TME. LMP1 has been shown to upregulate IL-10 through the p38/SAPK2 signaling cascade (46). About 60-68% LMP1-positive NPC tumors were found to express IL-10 (30, 73, 74). Interestingly, NPC-derived LMP1 could potentiate the function of CD4+ CD25+Foxp3+ Tregs by increasing the production of IL-10 (75). IL-10 acts on Tregs through IL-10R to maintain the expression and inhibitory activity of FOXP3 and to enhance the differentiation and immunosuppressive function of Tregs. Tregs are consistently detected in the circulation of NPC patients and EBV positive NPC tissues (76). Tregs are a suppressor T cell population which play an essential role in repressing the activation, proliferation and cytokine production of CD4+T cells and CD8+T cells. Tregs may also suppress B cells and dendritic cells. Tregs mediate their effects through secretion of immunosuppressive cytokines including TGFβ and IL-10 (77). Therefore, upregulation of IL-10 by LMP1 activates Tregs in NPC TME further protecting EBV-infected NPC cells from immune attack (**Figure 2**).

### NPC Derived Exosomes Containing LMP1 Can Promote Cancer Progression & Immune Evasion

EBV-infected lymphocytes and epithelial cells have been shown to release LMP1-containing exosomes (78). Furthermore, LMP1 containing exosomes have been identified in the serum of NPC patients and mice grafted with NPC tissues (79, 80). Interestingly, a strong positive correlation was observed between the levels of LMP1 and the exosomal marker, CD63, in NPC tissues (81). By nanoparticle tracking analysis and gradient purification, Hurwitz et al. showed that LMP1 not only increased the expression of exosomal protein markers but also stimulated exosome secretion (82). Exosomes are secreted microvesicles that bud directly from plasma membranes, which function in intercellular communication by transferring proteins, DNAs, mRNA and miRNAs to target cells to modulate their signaling pathways and cellular activities. Exosomes transfer information locally within the TME as well as systemically to distant tissue sites (31).

Previous studies have shown that exosomes released from LMP1 expressing C666-1 NPC cells and C33A cervical cancer cells could be effectively transferred to HUVEC cells and Rat-1 fibroblasts. In this study, exosomal-derived LMP1 was shown to be biologically active as it was capable of activating the PI3K/Akt and Erk/MAPK signaling pathways in recipient cells (80). LMP1 containing exosomes derived from NPC cells were also shown to directly induce anergy of T cells triggered by strong stimuli or mitogen-mediated TCR activation (33, 78). Collectively, these findings suggest that exosome-derived LMP1 can exert its oncogenic and immunosuppressive effects in non-EBV infected host cells within the TME to facilitate cancer progression.

FGF-2, EGFR, HIF1α; and galectin 9 (Gal-9) have also been found in NPC-derived exosomes, where they interact directly or indirectly with LMP1 (78, 80, 81, 83). Exosomal HIF1-α was found to be upregulated by LMP1 and shown to be functional in DNA-binding and transcriptional activities. Exosomal HIF1-α was found to modulate the expression of E- and N-cadherins, two junctional proteins associated with EMT, and to promote the recipient cells ability to migrate and invade (81). LMP1 was also found to promote the secretion of FGF2 and EGFR from exosomes (80, 83). Our previous studies have shown that LMP1 upregulates the FGF2/FGFR1 signaling pathway as well as HIF1α expression to promote aerobic glycolysis (65). FGF-2 is known as a potent angiogenic factor that participates in tumor invasion. EGFR, a downstream target of LMP1, promotes cell proliferation by activating multiple tyrosine kinase-associated signaling cascades. Exosomal EGFR had been shown to activate growth-promoting pathways: MAPK/ERK and PI3K/Akt in recipient cells (80). Accordingly, the interaction of LMP1 with FGF-2, EGFR and HIF1-α in exosomes promote cell proliferation, EMT, angiogenesis, and metastasis in recipient cells.

Interestingly, LMP1 was found to interact with Gal-9 in NPC-derived exosomes, even though Gal-9 is not recruited to lipid rafts by LMP1, nor is its expression induced by LMP1. Gal-9 is a β-galactoside binding lectin which has dual effects in controlling immune cells (84). Gal-9 acts as a pro-inflammatory factor that induces the maturation of dendritic cells, thereby promoting Th1 immune responses. Gal-9 is a ligand of Tim-3, a death-inducing receptor, that functions to self-limit immune responses. Gal-9 creates a negative feedback loop by inducing apoptosis of mature Th1 cells by binding to Tim-3, thereby terminating Th1 immune response (85). Gal-9 also promotes CD25+Foxp3+ Tregs differentiation and expansion (84). Gal-9 expression is induced by EBV infection and cytokines, particularly IFN*γ* and IL-1β (84, 86). NPC tumor cells and xenografted tumors were shown to express high levels of Gal-9, although its expression did not always correlate with that of LMP1 (78, 87). Gal-9-containing exosomes are commonly detected in the serum from NPC patients and mice grafted with NPC tissues. Exosomal delivery of Gal-9 has been shown to induce apoptosis in EBV-specific CD4+ T cells through Gal-9/Tim-3 interactions (88). Moreover, extracellular Gal-9 secreted from NPC cells stimulated CD33+ myeloid cells to produce IL-1β and IL-6, which tin turn promoted MDSC differentiation and expansion from CD33+ myeloid cells (89). Therefore, LMP1 containing exosomes secreted by NPC cells play an important role in modulation of the TME through altering stromal cell responses in proliferation, angiogenesis, invasion, migration, as well as immune surveillance. T cell proliferation was found to be inhibited by either recombinant LMP1 or Gal-9 with no synergy when these were combined (78). Given that LMP1 and Gal-9 have similar immunosuppressive properties, the impact on tumor immune evasion of LMP1 and Gal-9 in NPC-derived exosomes are worthy of further investigation.

### Upregulation of ISG15 in NPC Cells With Epithelial-Immune Dual Feature

In a recent study, single-cell transcriptome analysis identified a subpopulation of tumor cells expressing high levels of immune-related genes (27). Interestingly, the presence of cells with this “epithelial-immune” dual phenotype in NPC tissues were associated with a poorer prognosis. Their presence also correlated strongly with expression of co-inhibitory receptors (PD-1, LAG-3, TIM-3, TIM-4 and CD276) on CD8+ TILs, reflecting a more profound dysfunction of TILs in tumors containing this unique subpopulation of cancer cells. Functional studies also showed that the “epithelial-immune” tumor subpopulation displayed a more substantial capacity for tumorigenesis, a greater potential for immune cell recruitment and modulation, and was able to suppress IFN-*γ* production by T cells (27). Given that LMP1 expression in NPC cells upregulates many cytokines and immunomodulatory molecules that facilitate immunosuppression, the possible correlation between LMP1 expression and the presence of these “epithelial-immune” NPC cells is clear an area worthy of further investigation.

Epithelial-immune tumor cells expressed high levels of immune-related genes that included interferon (IFN)-response genes [e.g. IFN-stimulated gene 5 (ISG15)], major histocompatibility complex II (MHC II)-coding genes (e.g. HLA-DR), and complement genes (e.g. C3) (27). Among these genes, ISG15 is of particular note. ISG15 was initially associated with antiviral and antibacterial activities, and its expression is upregulated by IFNα and IFNβ as well as IFN regulatory factors (IRFs) such as IRF7 (90, 91). Interestingly, both ISG15 and IRF-7 are induced by LMP1 through activation of the NF-κB signaling pathway (92, 93). Reciprocally, IRF7 can bind to LMP1 promoter to induce LMP1 expression, forming a regulatory circuit with LMP1 (94). The possible correlation between IRF7-LMP1 positive feedback loop and ISG15 upregulation in NPC cells is also an area worthy of further investigation.

ISG15 is a ubiquitin-like protein which is present in cells as free ISG15 or in a conjugated form. Through the ISGylation pathway, ISG15 is conjugated to numerous cellular proteins involved in IFN-induced immune responses or in the regulation of cellular protein turnover. ISG15 has both immunomodulatory pro-tumor and anti-tumor functions. Intracellular ISG15 has been shown to promote the expression of MHC class I complexes in breast cancer cells, while secreted ISG15 has been shown to exhibit anti-tumor activities by inducing NK cell proliferation and migration towards tumor cells and the release of IFN-*γ* from lymphocytes (90, 95, 96). However, ISG15 has been shown to induce proliferation, apoptotic resistance, invasion and migration in cancer cells. Elevated expression of ISG15 has been demonstrated in a wide range of human tumors, where its overexpression correlated with differentiation, metastasis, and an unfavorable prognosis (90). In NPC patients, high levels of ISG15 expression are associated with local recurrence, poor overall survival (OS) and disease-free survival (DFS). Ectopic expression of ISG15 in NPC cells was accompanied by an increase of colony and tumorsphere formation *in vitro* and tumorigenicity *in vivo*. ISG15 was also found to increase the resistance of NPC cells to radiation and cisplatin treatment (97). Similar to ISG15, IRF7 also has dual functions. It is a key activator of IFN genes in response to viral infection. However, IRF7 itself is oncogenic, as it can promote anchorage-independent growth and malignant transformation of NIH 3T3 in athymic mice (98). IRF7 is commonly expressed in primary NPC tissues, where its expression correlates with that of LMP1 expression as well as cervical lymph node metastasis (99). IRF7 is involved in the maintenance of EBV latency and has a cooperative effect with LMP1 on cellular transformation (98, 100). It will be interesting to examine whether IRF7 and ISG15 have double-edged functions in NPC and whether they are involved in the oncogenic activities of LMP1. The involvement of IRF7 and ISG15 in modulating the inflammatory TME in NPC is clearly an area worthy of further investigation.

ISG15 overexpression in pancreatic cancer cells has been shown to confer resistance to gemcitabine (101), while ectopic expression in NPC cells reduced cell sensitivity to radiation and cisplatin treatment (102). However, completely opposite effects were observed in oesopharyngeal cancer cells, where siRNA-mediated depletion of ISG15 or treatment with cytotoxic drugs (5-Fu and rapamycin), resulted in an increase in endogenous autophagy and an enhancement of drug-resistant cancer cells (103). In line with these findings, reduced ISG15 levels were observed in drug-resistant cancer cell lines and ISG15 overexpressing breast tumor cells were shown to have heightened sensitivity to the anticancer drug camptothecin (Topotecan) (104). These contradictory findings suggest that ISG15 expression in the tumor could be a factor affecting chemotherapy treatment of cancer and the contribution of ISG15 in tumor chemosensitivity may be tissue specific. Therefore, further investigations are necessary before considering employing ISG15 as a novel therapeutic target in NPC.

### LMP1 Promotes Cancer Cell Growth, Survival, EMT, Angiogenesis, and Metastasis by Modulating Stromal Cells Within the TME

When expressed in epithelial cells, LMP1 induces an epithelial-mesenchymal transition (EMT), by stimulating a process termed cadherin-integrin “switching”. This results in the downregulation of E-cadherin, *γ*−catenin and Mucin 1, and the concomitant upregulation of mesenchymal markers, vimentin, N-cadherin, fibronectin, Twist, Snail and matrix metalloproteinase (MMPs), all of which cooperate to promote an EMT. Collectively, this transition promotes epithelial cell migration and invasion, which can facilitate cancer metastasis (8, 105). LMP1 can also promote angiogenesis through induction of pro-angiogenic factors such as VEGF, FGF and IL-8. The induction of cell growth by LMP1 is mediated through upregulation of many growth factors such as EGFR and cytokines such as IL-6 (8, 105). LMP1 can also promote cell survival through upregulation of anti-apoptotic proteins including Bcl-2 and A20 to protect infected cancer cells from potentially cytotoxic signals such as Fas ligand (FasL), TNF-related apoptosis-inducing ligand (TRAIL) mediated by cytotoxic T cells (8, 31).

While the aforementioned phenomenon relates to the effects of LMP1 in EBV-infected epithelial tumor cells, it is possible that LMP1-induced growth factors, cytokines and exosomes, serve to modulate the phenotype of other cell types within the TME. These various tumor-derived factors not only influence leukocytes, but also impact on the behavior of other cell types such as fibroblasts, mesenchymal cells and endothelial cells. In recent years, research has focused on a subset of fibroblasts termed cancer-associated fibroblasts (CAFs), which are activated in response to wound healing or chronic inflammation (106). CAFs are activated fibroblasts in the TME that have a distinct immune-phenotype compared to quiescent fibroblasts. Fibroblasts function as a source of extracellular matrix (ECM) and a play an important role during wound healing, inflammation, and tissue repair. In response to tumor-derived factors (TGF-β, LPA, IL-1, IL-6) or tumor-derived exosomes, which activate the SMAD, JAK-STAT and NF-κB pathways, fibroblasts become activated. These then participate in tissue remodeling through the section of growth factors (TGF-β, HGF, VEGF), cytokines (IL-6, CCL2, CXCL12), and ECM, which impact on surrounding epithelial cells and immune cells. Indeed, a large body of evidence now supports a role for CAFs in maintaining an immunosuppressive TME by modulating the activity and function of CD8+ve T cells, Treg cells and TAMs (106).

Numerous studies attest to the role of cancer-associated fibroblasts (CAFs) in the pathology of epithelial malignancies. CAFs promote tumor progression through the secretion of growth factors, cytokines and proteases, that not only stimulate cell proliferation but degrade the extracellular matrix, allowing tumor cell to migrate and invade the surrounding stroma (107, 108). Although research into CAFs and NPC are limited, several recent studies have identified the presence of CAFs in NPC tissue biopsies. Using α-smooth muscle actin (α-SMA) as an established CAF marker, several immunohistochemical-based studies have identified the presence of CAFs in EBV-positive NPC (109, 110). In their study, Wang et al. described a correlation between the degree of CAF positivity and expression of the CXCR4 ligand, SDF-1 (CXCL12) on NPC tumor cells (109). Moreover, a striking correlation was observed between the levels of α-SMA and the endothelial cell marker CD34, a marker of endothelial cells and microvessel density, supporting a role for CAFs in NPC progression. The study by Chen et al. established that CAF density correlated with a poor prognosis. Here, Cox multivariate analysis revealed that CAF density could be used as an independent prognostic factor to predict patient survival (110).

To date, these studies have not examined differences between LMP1 positive cases of NPC. However, an *in vitro* study has shown that extracellular vesicles (EV’s) loaded with LMP1 were able to induce the formation of CAFs from normal fibroblasts through a process involving NF-κB signaling (111). This preliminary finding raises the possibility that LMP1-positive tumors may be more efficient at inducing CAF formation.

## Strategies for LMP1-Based Therapeutics in EBV-Positive NPC Tumors

NPC is a highly radiosensitive tumor, and intensity-modulated radiotherapy (IM-RT) is the standard treatment for primary tumors and regional lymph node metastases. For advanced disease or tumors associated with cervical lymph node metastasis, concurrent chemotherapy-RT is applied. Although the overall survival rates of patients with early disease are favorable, treatment failure and distant metastasis still occur in a significant number of patients. Also, a large fraction of patients suffer long-term post-treatment toxicities and a poor quality of life (4). The limitations of current treatment protocols along with the significant number of patients who present with advanced disease advocate the development of more effective therapies that improve the duration of response and survival for patients with metastatic NPC.

LMP1 has multiple oncogenic functions that can impact on cancer development. The strong association of EBV with NPC, coupled with the fact that a significant proportion of tumors express the LMP1 oncoprotein, has led to the development of numerous approaches to target LMP1 as a potential therapeutic for EBV-driven malignancies (**Table 1**).

**Table 1:** Strategies for LMP1-based therapeutics in EBV-positive NPC tumors.

Therapy	Principal	Experimental/Clinical Results	Ref.
***Vaccine-based immunotherapy***	EBV-specific vaccine for enhancing immune response in patients with EBV-associated malignancies.	With recombinant poxvirus vaccine encoding a polyepitope containing multiple HLA A2-restricted LMP1 epitopes, strong CTL responses were induced. The outgrowth of LMP1 expressing tumors in HLA-A2/Kb mice were repressed.	(112)
		An EBV antigen-based scramble-antigen vaccine (SAVINE) incorporating random overlapping peptide sets from EBNA1, LMP1 & LMP2 activated all immune cells including Th1 cells and CTLs against these three viral latent proteins. This vaccine also activated LMP1 & LMP2 responses in healthy individuals & NPC patients.	(113)
		Autologous DCs were transduced with vaccine: an adenovirus encoding a truncated LMP1 and full length LMP2 (Ad-ΔLMP1-LMP2). In a phase II trial, vaccine transduced DCs could be generated and safely administered to NPC patients with extensive disease. No significant toxicity was observed.	(114)
***Adoptive cell transfer Immunotherapy***	Infusion with autologous EBV-specific CTLs.	Simulation of PBMCs with LMP1 43aa N-terminal deleted mutant expressing DCs resulted in a thousand-fold induction of LMP1-specifiic CTLs capable of lysing EBV-infected NK lymphoma.	(115) (116)
		With autologous CTLs enriched for specificity to LMP1& LMP2 Ags, durable clinical response without toxicity in heavily pre-treated patients with EBV-associated lymphoma was observed. In patients with recurrent NPC, regression of the majority of pulmonary lesions were indicated.	(117) (118)
		With adenoviral vaccine encoding epitopes from LMP1 & LMP2 as a polyepitope (Ad5F5-LMPpoly), strong LMP1-specific CTLs were generated. Also, LMP1-expressing NPC growth in mice was inhibited.	(119)
		With adenoviral vaccine encoding EBNA1 linked to multiple CD8 T-cell epitopes from LMP1 & LMP2 (AdE1-LMPpoly), stimulated CD8+ T cells specific for LMP1, LMP2 & EBNA1 expanded effectively and recognized EBV latency II HL cells.	(120)
		AdE1-LMP poly adenovirus-based adoptive immunotherapy resulted in expansion of EBV-specific T cells. Overall survival of NPC patients were increased with mild toxicities.	(121)
***Antibody-based Immunotherapy***	Infusion with LMP1-specific antibodies	LMP1 specific Abs generated by immunizing rabbit with peptides representing LMP1 extracellular loop domains. Abs could mediate complement-driven cytolysis in ~ 35%–55% of EBV-infected B lymphocytes.	(122)
		Abs generated from peptides from two extracellular loops of LMP1 (2LS peptides) have remarkable antitumor activities in mice.	(123)
		An immunoconjugate called HLEAFab-MCC was generated by conjugating mitomycin with human Ab Fab fragment specific for LMP1 extracellular domains. This immunoconjugate killed LMP1 positive NPC cells in culture and also inhibited the growth of NPC xenografts in nude mice.	(124)
***Immune checkpoint inhibitor***	Targeting PD1 or PD-L1 with monoantibody	KEYNOTE-028 multicohort phase 1b trail study: Pembrolizumab (mAb against PD-L1) monotherapy in patients with recurrent or metastatic NPC. Pembrolizumab had promising anti-tumor activity and a manageable safety profile.	(125)
		A Phase II study (NCI-9742) investigating the anti-tumor activity of nivolumab (an anti-PD1 mAb) in patients with multiply pretreated recurrent NPC. Better responses without unexpected toxicity were observed in patients bearing PD-L1 positive tumors.	(63)
		A phase I trial investigating camrelizumab (an anti-PD1 Ab) monotherapy in patients with metastatic NPC. Promising response with 59% 1-year overall survival (OS) was observed. A combination of gemcitabine, cisplatin and camrelizumab resulted in the best clinical outcome for patients with metastatic NPC although unacceptable high rate of grade >3 adverse events was observed.	(126)
***RNA-cleaving DNAzyme***	DNAzymes: a synthetic ssDNA engineered to bind & cleave its targeted mRNA molecules.	The LMP1 sequence-specific DNAzymes significantly reduced LMP1 expression resulting in G0/G1 arrest and growth inhibition in EBV-positive B95.8 cells.	(127)
		DZ509, a specific LMP1 targeted DNAzyme inhibited proliferation, apoptotic resistance, and xenograft tumor growth in LMP1 expressing NPC C666-1 cells.	(128)
		NPC patients were injected intra-tumorally with DZ1 (an LMP1-targeting DNAzyme) & received radiation therapy. Average tumor regression rate and microvascular permeability were significantly improved.	(129)
***Peptide therapy***	Peptide-based inhibitors bind to EBV latent proteins	The B1.12 peptide (ACPLDLRSPCG) which selectively bind to first extracellular loop at the N-terminal domain of LMP1 downregulated A20, repressed NF-κB and Akt signaling, induced G0/G1 arrest & reduced viability in the EBV-positive NPC C666-1 cells.	(130)
	LMP1-EBNA1 dual targeting peptide conjugated with nanoparticles	Dual-targeting peptide-guided approach for precision delivery & cancer monitoring by using a safe upconversion nanoplatform. Targeting LMP1 assisted *in vivo* drug delivery & *in vitro* cellular uptake, contributing to tumor-specificity. The presence of pH-sensitive and cleavable linker contributes to EBNA1-LMP1 binding peptides release from the nanoparticles in acidic pH recapitulating the tumor microenvironment.	(131)

### T Cell-Based Immunotherapy: Vaccine-Based Immunotherapy

An EBV-specific vaccine which aims to enhance the immune response in patients with EBV-associated malignancies has been evaluated. A preclinical study reported that a recombinant poxvirus vaccine encoding a polyepitope protein containing multiple HLA A2-restricted LMP1 CTL epitopes, not only induced a strong CTL response but could also repress the outgrowth of LMP1-expressing tumors in HLA-A2/Kb mice (112). However, given that the LMP1 epitopes were HLA-A2-restricted, the vaccine is not suitable for all ethnic groups. Therefore, a range of HLA allele –restricted epitopes are necessary for this approach to be universally applicable. In another study, Lutzky et al. generated an EBV antigen-based scramble-antigen vaccine (SAVINE) which incorporates random overlapping peptide sets from EBNA1, LMP1, LMP2. These peptide sets were then inserted into a replication-deficient adenovirus to construct Ad-SAVINE. This Ad-SAVINE provides a platform to activate all possible immunological cells, including helper cells and CTLs against the three viral latency proteins. This Ad-SAVINE formulation was also capable of activating LMP responses in healthy individuals as well as NPC patients (113).

In a phase II clinical study, Chia et al. evaluated a dendritic cell (DC) vaccine to target LMP1 and LMP2. The autologous DCs were transduced with an adenovirus encoding a truncated LMP1 and full-length LMP2(Ad-ΔLMP1-LMP2). Ad-ΔLMP1-LMP2 transduced DCs could be successfully generated and safely administered to 16 NPC patients with extensive disease; fortunately, no significant toxicity was observed in any patient. However, nine out of 12 patients developed a delayed-type hypersensitivity response, and only three out of 12 patients had a clinical response. Although these DCs activated LMP1/2 specific T cells *in vitro*, there was no significant increase in the number of peripheral LMP1/2-specific T cells (114). Further exploration in a modification of the DC vaccine or combination with other therapeutic interventions may be necessary to increase the vaccine therapeutic effectiveness

### T Cell-Based Immunotherapy: Adoptive Cell Transfer Immunotherapy

Accumulating clinical evidence indicates that adoptive cell transfer immunotherapy (ACT) is a safe and effective approach for treating EBV-associated malignant diseases. ACT is an approach where patients are infused with autologous EBV-specific CTLs expanded *ex vivo* by repeated stimulation with EBV-transformed LCLs. ACT is particularly effective in malignancies displaying an EBV latency III program such as post-transplant lymphoproliferative disease (PTLD), where the full repertoire of EBV latency-associated proteins are expressed (132). However, in EBV latency II associated-malignancies, including NPC and HL, only a fraction of patients receiving ACT showed clinical responses (133, 134, 135). This lack of clinical responsiveness may be due to the restricted pattern of viral latent antigen expression in cancer cells, the poor immunogenicity of EBV proteins (LMP1, LMP2 and EBNA1) expressed in NPC cells and a local immunosuppressive TME. As only a minor fraction of the CTLs generated from LCLs recognize the EBV proteins expressed in NPC (136, 137), protocols designed to specifically stimulate LMP1, LMP2, and EBNA1 CTLs are necessary.

The generation of LMP1 specific CTL clones has proved difficult given the toxicity associated with high-levels of LMP1 expression (115). This toxicity has limited the use of DCs as APCs for CTL stimulation. Therefore, LMP1-specific CTLs are commonly generated using LMP1-derived peptides or APC’s infected with a recombinant vaccinia virus overexpressing LMP1 (137). In these approaches, a step of T cell cloning is necessary. However, the generation of T cell clones is labor-intensive, limiting the use of LMP1 specific CTL for ACT. In this regard, Gottschalk et al. developed a new protocol that generated polyclonal LMP1 specific CTL lines by stimulating PBMCs with DCs expressing a functionally inactive and non-toxic LMP1 mutant in which the 43 amino acid N-terminus was deleted. Simulation of PBMCs with DCs expressing this LMP1 mutant resulted in a thousand-fold induction of LMP1-specific CTLs (115). Moreover, these polyclonal populations were shown to be functional, as they were able to kill an EBV-infected NK cell line and NK lymphomas, respectively (116). Promising results were obtained in clinical trials evaluating the efficacy of polyclonal CTLs enriched for the LMP1 and LMP2 antigens, where durable clinical responses without significant toxicity were observed in heavily pre-treated patients with EBV-associated lymphoma (117). Furthermore, in a clinical study of NPC, adoptive transfer of autologous CTLs specific for LMP1 and LMP2 epitopes in a patient with recurrent NPC, resulted in regression of the majority of pulmonary lesions, although the primary tumor did not wholly regress (118). These findings suggest that CTLs, which specifically target LMP1 and LMP2 antigens, may be useful in targeting tumor-associated viral antigens in patients with EBV latency II-associated malignancies.

To enhance the specificity of CTLs and their anti-tumor response for EBV latency II-associated malignancies, Duraiswamy et al. developed a replication-defective adenoviral vaccine encoding 13 distinct HLA class I-restricted CTL epitopes from LMP1 and LMP2 as a polyepitope. This recombinant adenovirus LMP polyepitope (Ad5F5-LMPpoly) vector, stimulated robust CTL responses towards multiple LMP1 and LMP2 antigens. The expanded T cells were able to kill autologous target cells sensitized with LMP1 or LMP2 CTL antigens *in vitro*. In an *in vivo* mouse model, this polyepitope vaccine consistently generated strong LMP1-specific CTL responses and was capable of inhibiting the growth of an LMP1-expressing NPC tumor (119). Furthermore, Smith et al. developed an additional replication-deficient adenoviral vaccine that encoded EBNA1 linked to multiple CD8 T-cell epitopes from LMP1 and LMP2. This recombinant adenoviral EBNA1-LMP polyepitope (AdE1-LMPpoly) technology appeared effective in expanding CD8+ T cells specific for LMP1, LMP2A, and EBNA1. The expanded T cells could also efficiently recognize HL cells which expressed a limited array of EBV Latency II antigens (120). This adenovirus-based adoptive immunotherapy has been evaluated in a Phase I study involving NPC patients with recurrent and metastatic diseases. In this study, EBV-specific T cells were successfully expanded from 16 of 22 patients, with only mild toxicities such as grade 1 flu-like symptoms being observed in 14 patients. Furthermore, an increased overall survival from 220 to 523 days was observed in treated patients when comparing to the control patients who did not receive ACT (121). These studies demonstrate that replication-incompetent adenoviral vaccine technology is a successful protocol in the expansion of CTLs, specifically targeting multiple LMP1, LMP2, and EBNA1 epitopes. Clinical studies also proved that this approach is safe and effective in improving outcomes for patients with relapsed or metastatic diseases. Clinical studies to further determine the efficacy of this approach are in the development.

### Antibody-Based Immunotherapy

The extracellular loops of LMP1 are generally poorly immunogenic. Paramita et al. generated LMP1 specific antibodies by immunizing rabbit with peptides encompassing domains of the LMP1 extracellular loops. These antibodies could mediate complement-driven cytolysis in approximately 35%–55% of EBV infected B lymphocytes. Although only marginally immunogenic, LMP1 peptide-specific immunization may have a therapeutic potential for patients carrying LMP1 expressing tumor (122). In another study, Delbende et al. reported that conformational peptides mimicking two extracellular loops of LMP1 (2LS peptides) could induce high-affinity antibodies. These antibodies were shown to have remarkable anti-tumor activities in mice. Interestingly, these 2LS peptide constructs could not be recognized by sera from EBV-seropositive individuals or NPC patients, suggesting that antibody responses with these specificities were not produced as a result of physiological immune responses in EBV-infected individuals (123). Therefore, this vaccination strategy may be useful in eliciting an immune response to target LMP1 expressing tumor cells in NPC patients. The combination of this antibody approach with radiotherapy or chemotherapy may improve overall clinical outcome. Indeed, Chen et al. developed a novel human antibody Fab fragment specific for the LMP1 extracellular domains, which was subsequently conjugated with the chemotherapeutic agent, mitomycin C, to generate an immunoconjugate (HLEAFab-MCC). This immunoconjugate killed LMP1 positive NPC cells in culture and also inhibited the growth of NPC xenografts in nude mice, indicating its therapeutic potential in the treatment of NPC (124).

### Immune Checkpoint Inhibitor

PD-L1, which is a putative target of LMP1, is commonly expressed on NPC tumor cells. Binding of PD-L1 to its counter receptor, PD-1, on T cells, elicits a strong immunosuppressive effect, blocking the activity of EBV-specific CTLs. In this regard, immune checkpoint inhibitor therapy aims to block inhibitory PD-L1/PD-1, signaling to reactivate the anti-tumor function of infiltrating cytotoxic T cells. Several Phase I-II trials using monoclonal antibodies against checkpoint proteins have shown that targeting PD1 or PD-L1 with monoclonal antibodies is a viable therapeutic invention for patients with recurrent or metastatic NPC (137, 138). In the KEYNOTE-028 multicohort phase 1b trial study, pembrolizumab monotherapy, a monoclonal antibody specific for PD-L1, was evaluated in a cohort of NPC patients (n=27) with PD-L1 positive, recurrent or metastatic NPC. Over a median follow-up of 20 months, an objective response rate (ORR) of 25.9% was obtained. Also, drug-related adverse events occurred in 15% of patients, and Grade ≥ 3 drug-related adverse events were reported in 29.6% of patients. These findings demonstrated that pembrolizumab had promising anti-tumor activity and a manageable safety profile (125). Furthermore, in a multi-national Phase II study (NCI-9742), the anti-tumor activity of nivolumab (a humanized anti-PD1 monoclonal antibody) in patients with multiply pre-treated recurrent or metastatic NPC (n=44), was evaluated. Promising results were obtained in this study, with antibody-treated patients achieving an overall ORR of 20.5% and 1-year overall survival (OS) rate of 59%. Significantly better responses were observed in the proportion of patients who were diagnosed with PD-L1 positive tumors. An additional positive outcome was the finding that no unexpected toxicities to nivolumab were reported in the study (63).

A phase I trial investigating the efficacy of camrelizumab, a humanized anti-PD1 antibody, in patients with metastatic NPC, 9/45 patients (ORR 20.5%) achieved favorable response with 59% 1-year overall survival (OS). In addition, the effectiveness of camrelizumab monotherapy was further enhanced by combination with chemotherapy (126). In the same trial study, the combination of gemcitabine and cisplatin with camrelizumab resulted in the best clinical outcome for patients with metastatic NPC with an ORR of 91% and 1-year PFS of 68%. However, an unacceptably high rate of grade >3 adverse events (87%) was observed (126). Overall, clinical trial results suggest that anti-PD1/PD-L1 therapy is a potential therapeutic for patients with recurrent or metastatic NPC. Further phase I-III trials for evaluation of clinical outcomes, optimization of drug combinations and determination of safety profiles, are in progress.

### RNA-Cleaving DNAzyme

DNAzymes are synthetic, single-stranded catalytic DNAs that are engineered to bind and cleave their mRNA target molecules. DNAzymes have been developed as a potential therapeutic tool to inhibit LMP1 gene expression in malignant EBV infected tumor cells. Lu et al. firstly reported the successful knockdown of LMP1 with DNAzymes in EBV positive B95.8 LCLs, where LMP1 is constitutively expressed. These LMP1 sequence-specific DNAzymes significantly downregulated LMP1 expression, induced G0/G1 arrest and also inhibited the proliferation of B95.8 cells (127). Furthermore, Ke et al. reported that DZ509, a specific LMP1 targeted DNAzyme, was capable of inhibiting proliferation and inducing apoptosis in C666-1, an EBV positive NPC cell line which constitutively expresses a weak level of LMP1 (128). DZ509 also significantly suppressed C666-1 xenograft growth in nude mice. In another clinical study, the anti-tumor and radiosensitizing effects of DZ1, an LMP1-targeting DNAzyme, were evaluated in NPC patients. Forty NPC patients received intra-tumoral injection with DZ1 along with radiation therapy. After a 3-month follow up, the average tumor regression rate and microvascular permeability were improved significantly in DZ1-treated patients compared to controls. An additional positive outcome from this study was the lack of adverse events being reported when DZ1 therapy was combined with radiotherapy. Results from this study suggest a potential of using DZ1 as a radiosensitizer for the treatment of NPC (129). However, there are still hurdles to overcome in terms of improving method of drug delivery, such as exploring the use of nanoparticles for local and systemic administration. Nevertheless, the ORRs observed in these early studies are disappointingly consistent with other tumors and suggest that more effective response might be obtained by combining immune checkpoint inhibitor therapy with other approaches such as ACT.

### Peptide Therapy

Strategies that use peptide-based inhibitors to reactivate EBV lytic replication or inhibit the oncogenic function of EBV latent proteins is being explored as a novel therapeutic approach to cancer management. Several peptides that directly target EBNA1 have been developed and demonstrated to effectively block EBNA1 homodimerization or inhibit EBNA1 interaction with the OriP sequence of the EBV genome, preventing EBNA1-mediated replication and transcription (130–141). One of the inhibitors, VK-2019, is now engaged in a Phase I/IIa clinical trial for EBV-positive NPC patients (142). In addition to EBNA1, attempts have been made to target the LMP1 protein by peptide inhibitors. In a recent study, Ammous-Bou et al. identified a novel peptide called B1.12 (ACPLDLRSPCG) from a phage display peptide library. This B1.12 peptide was demonstrated to selectively bind to the first extracellular loop at the N-terminal domain of LMP1. B1.12 was shown to downregulate A20 expression, repress NFκB and Akt signaling, induce G0/G1 cell cycle arrest and reduce cell viability in C666-1 NPC cells (130). Given that LMP1 mediates its cytoplasmic C-terminal CTAR domains to activate various signaling pathways for its oncogenic activities, it is not clear how the B1.12 peptide, which targets the N-terminal extracellular loop of LMP1 interferes LMP1-mediated signaling and oncogenic activities, and this is necessary to be further investigated.

Recently, our group has established an EBNA1-LMP1 dual targeting agent UCNP-P5. The EBNA1-LMP1 binding peptide conjugated with the lanthanide upconversion nanoparticles NaGdF4:Yb3+, Er3+@NaGdF4 (UCNP), as to enhance the stability, prolong fluorescent lifetimes, and minimize interference by biological autofluorescence. The nanoparticle and the peptide were connected with a pH-sensitive and -cleavable linker; the EBNA1-LMP1 binding peptides can be released from UCNP in acidic pH recapitulating the tumor microenvironment. Intravenous injection was used to deliver these agents and the animal results showed that UCNP-P5 could significantly reduce the average tumor size. Targeting of LMP1 can assist *in vivo* drug delivery and *in vitro* cellular uptake, which contribute to the tumor-specificity; the anti-EBNA1 functions were still maintained (131).

## Concluding Remarks

NPC is characterized by the presence of an abundant lymphoid and stromal infiltrate. The anti-tumor function of the CTLs in NPC is believed to be suppressed by cytokines, immune checkpoint proteins, and cancer-derived exosomes present in the TME (10, 26, 27, 31, 40). EBV-encoded LMP1 functions as a constitutively active TNF receptor. Through its C-terminal CTAR1 and CTAR2 domains, LMP1 activates multiple signaling pathways. It regulates the expression of various downstream targets associated with cell growth, survival, EMT, migration, invasion, aerobic glycolysis as well as immune evasion (8, 31). LMP1 has immunomodulatory properties as it regulates the expression of many cytokines, chemokines, adhesion molecules, antigen processing and presentation proteins, all of which modulate the immune responses of TILs in TME (8, 31). For example, LMP1 induces IL-18 and IP-10 to recruit and suppress NK and T cells. Induction of PD-1 by LMP1 suppresses the anti-tumor activities of TILs (8, 31). The induction of aerobic glycolysis by LMP1 leads to increased secretion of IL-1β, IL-6, IL-18, and GM-CSF, which in turn promotes MDSC differentiation and expansion as well as cancer cell proliferation and resistance to apoptosis (67) (**Figure 2**). In addition, LMP1-containing exosomes released by NPC cells induces anergy in activated T cells, while FGF-2, EGFR, HIF-a, and Gal-9 in LMP1-containing exosomes promote proliferation and metastasis as well as immune exhaustion in recipient cells (33, 78, 105). LMP1 has multiple oncogenic functions and therefore is a critical therapeutic target.

Many research groups have attempted different approaches to targeting LMP1 for therapeutic use (**Table 1**). Several recombinant adenovirus or poxvirus vaccines which encode different CTL epitopes from LMP1, LMP2, and EBNA1 as a polyepitope have been established. Clinical studies have proved the safety and effectiveness of these vaccines (119–121). Furthermore, antibodies specifically targeting the extracellular loops of LMP1 for therapeutic use have been generated. The LMP1 specific antibody conjugated with mitomycin C (HLFAFab-MCC) has been shown to kill NPC cells *in vitro* and *in vivo* (124). Given that PD-L1 immune checkpoint protein induced by LMP1 is highly expressed on NPC tumor cells, several Phase I-III clinical studies have been performed, which aim to target the PD-1/PDL-1 pathway in patients with advanced NPC. Further clinical trials for evaluation of clinical outcomes, drug combinations, safety determination are in progress (137). The use of DNAzymes to knock down LMP1 also has therapeutic potential. DZ509, a specific LMP1 targeted DNAzyme, has been shown to suppress the growth of the NPC tumor cells in culture and nude mice significantly (128).

Furthermore, one peptide specifically targeting LMP1 N-terminal extracellular loop domain has been shown to inhibit LMP1-mediated NFκB and Akt signaling. This peptide probes also reduced NPC cell viability and proliferation (130). Our group has developed EBNA1-specific fluorescent peptide probes (L2P4 & UCNP-P4) which target the EBNA1 dimerization site (139, 140). These probes have proved successful in the imaging of tumor xenografts *in vivo* and also in suppressing the growth of EBV-positive tumor cells *in vitro* and *in vivo*. A similar approach may also be applied to the LMP1 protein. LMP1-specific peptides conjugated with an appropriate fluorophore may be useful in both the tracking of LMP1 containing exosomes and LMP1-expressing tumors *in vivo* as well as inhibiting LMP1 functions. EBNA1 functions to maintain EBV latency enhance replication and transcription of EBV genome. It will be interesting to investigate whether combinations of EBNA1- and LMP1-specific peptide probes to inhibit EBV latency and LMP1-mediated tumorigenesis might prove useful for the treatment of EBV-associated malignancies. Our newly made EBNA1-LMP1 dual target agent has shed some lights to this aspect, thus, LMP1 represents another attractive EBV target for us to pursue (131).

Over the last 50 years we have learnt that combinations of chemotherapeutic drugs are the most effective way of treating cancer. This is also the case with contemporary biological therapies where various anti-cancer vaccines and agents will only be effective if combined with interventions that target the TME. In this regard NPC provides a useful paradigm to explore the impact of combinatorial therapies that target tumor-specific proteins such as LMP1 while also blocking the immunosuppressive and tumor promoting effects of the TME.

## Author Contributions

AL, CD, and HL wrote the manuscript. K-LW and LY critically reviewed the manuscript. All authors contributed to the article and approved the submitted version.

## Funding

This work was supported by Research Grants Council of Hong Kong (Grant No. HKBU12300117).

## Conflict of Interest

The authors declare that the research was conducted in the absence of any commercial or financial relationships that could be construed as a potential conflict of interest.
